# Pulvis Doveri

**Published:** 1882-12

**Authors:** 


### Pulvis Doveri.

People whose "inward griefs and peristaltic woes" have been relieved by the powder of Dover, do not generally know to whom they are indebted for this excellent compound. Doctor Dover was a friend and probably pupil of the great Sydenham. He commenced practice in Bristol, where, having made some money, he longed to make more. The Roll of the College of Physicians tells us that he joined with some merchants in fitting out two privateers for the South Seas, in one of which, the "Duke," he himself sailed from Bristol, August 2, 1708. On the passage out they touched at the Island of Juan Fernandez, where Dover, on the 2d of February, 1709, found Alexander Selkirk, who had been alone on the island for four years and four months, and whom Dover brought away in the "Duke." In the April following, Dover took Ginaguil, a city or town of Peru, by storm. In December, 1709, the two privateers took a large and valuable prize, a ship of 20 guns and 190 men, in which Dover removed from the "Duke," taking Alexander Selkirk with him as master, and finally reaching England in October, 1711. After this cruise Dr. Dover removed to London, where his practice soon became great. His patients, and the apothecaries who wished to consult him, addressed their letters to the Jerusalem coffee house, where, at certain hours of the day, he received most of his patients.—*Can. Jour. of Med. Science.*

